# Polymer-Based Additive Manufacturing in Cervical Cancer Management: A Review

**DOI:** 10.3390/polym18202455

**Published:** 2026-10-09

**Authors:** Xiaolong Wang, Nanshan Lin, Yiqiong Cao, Jaydeep Vishwakarma, Xianhu Liu, Chenchen Ren

**Affiliations:** 1 Tianjian Laboratory of Advanced Biomedical Sciences, Zhengzhou University, Zhengzhou 450002, China; 2 Équipe de Recherche sur les Processus Innovatifs (ERPI), Université de Lorraine, F-54000 Nancy, France; 3 National Engineering Research Center for Advanced Polymer Processing Technology, Zhengzhou University, Zhengzhou 450002, China; 4 Zhengzhou Key Laboratory of Cervical Diseases, Zhengzhou 450052, China; 5 National Clinical Research Center for Obstetrics and Gynecology, Henan Branch, Zhengzhou 450052, China

**Keywords:** cervical cancer, polymer-based additive manufacturing, 3D printing, brachytherapy, therapeutic scaffolds, local drug delivery, bioinks, tumor models, anatomical models, gynecologic oncology

## Abstract

Cervical cancer management increasingly requires patient-specific and locally adaptable technologies to address complex pelvic anatomy, heterogeneous tumor geometry, limited local drug retention, and the need for improved planning and education. Polymer-based additive manufacturing offers a promising strategy by integrating digital design, customizable three-dimensional geometry, and tunable material properties. This review summarizes recent progress in polymer-based additive manufacturing for cervical-cancer-related applications from a material–process–structure–function perspective. Four application domains are discussed: 3D-printed polymeric devices for brachytherapy assistance, therapeutic scaffolds and local delivery systems, bioprinted research platforms, and patient-specific anatomical models for education, planning, and communication. In brachytherapy, printable templates and applicator accessories can improve anatomical adaptation, needle guidance, and procedural reproducibility. In therapeutic scaffolds, polymers enable local retention, controlled release, mechanical support, mucoadhesion, and mucosal interface regulation. In bioprinted tumor models, hydrogel bioinks provide controllable three-dimensional microenvironments for studying spheroid formation, epithelial–mesenchymal transition, drug response, oxygen gradients, and molecular diffusion. Patient-specific anatomical models further support spatial understanding, procedural training, multidisciplinary communication, and patient-centered decision-making. Future progress will depend on standardized workflows, clinically relevant validation, material safety evaluation, and integration with personalized oncology.

## 1. Introduction

Cervical cancer remains a major global health challenge and is among the most common malignancies affecting women worldwide. According to recent global cancer estimates, more than 660,000 new cases and approximately 350,000 deaths were attributed to cervical cancer in 2022, highlighting its persistent contribution to cancer-related morbidity and mortality [1]. Persistent infection with high-risk human papillomavirus is the principal etiological driver of cervical carcinogenesis, which typically evolves through a stepwise precancerous phase before progressing to invasive disease [2,3]. Although cervical cancer is largely preventable through prophylactic human papillomavirus (HPV) vaccination, population-based screening, and timely treatment of precancerous lesions, its burden remains unevenly distributed, with the majority of deaths occurring in low- and middle-income regions [1,4]. These disparities are further compounded by regional differences in healthcare infrastructure and access to specialized treatment technologies, including advanced radiotherapy and image-guided brachytherapy. Therefore, cervical cancer represents not only a preventable malignancy but also a clinically complex disease in which early detection, precise local intervention, individualized treatment planning, and long-term preservation of female health remain central therapeutic concerns.

**Figure 1:**
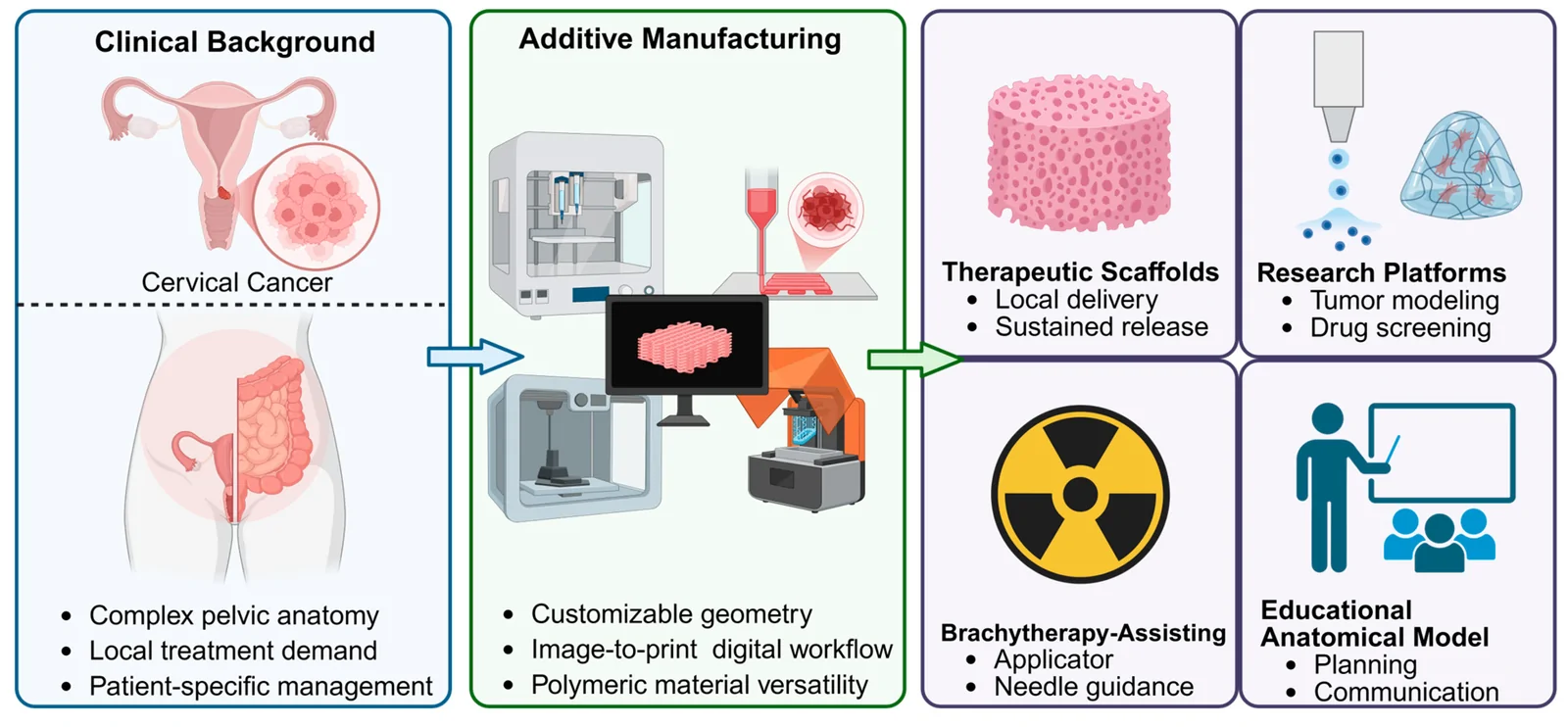
Overview of polymer-based additive manufacturing for cervical-cancer-related applications. Cervical cancer management is challenged by complex pelvic anatomy, local treatment demands, and patient-specific clinical needs. Polymer-based additive manufacturing enables customizable geometry, image-to-print digital workflows, and material versatility, supporting therapeutic scaffolds, bioprinted research platforms, brachytherapy-assisting devices, and educational anatomical models. Created in BioRender. Lin, Nan. (2026) https://BioRender.com/r59ddqx.

Despite substantial advances in HPV vaccination [5], screening [6,7], surgery [8,9], radiotherapy [10], chemotherapy [11], targeted therapy [12,13], and immunotherapy [14], the clinical management of cervical cancer still faces several practical limitations. As shown in Figure 1, cervical cancer arises in an anatomically complex pelvic region where the cervix, vagina, uterus, parametria, bladder, rectum, and pelvic sidewall are closely interrelated [15]. Tumor size, stromal invasion, parametrial extension, vaginal involvement, and postoperative anatomical distortion vary markedly among patients, making standardized therapeutic devices and conventional two-dimensional planning approaches insufficient in selected clinical scenarios [16]. This limitation is particularly relevant to brachytherapy, where commercial intracavitary applicators or freehand interstitial needle implantation may fail to achieve optimal geometric adaptation, reproducible needle guidance, and adequate target coverage in patients with bulky tumors, parametrial invasion, vaginal stenosis, or distorted pelvic anatomy [17]. Second, systemic chemotherapy and radio sensitizing agents remain important components of cervical cancer treatment, but their clinical use is limited by off-target exposure and treatment-related toxicities, including nephrotoxicity, myelosuppression, gastrointestinal toxicity, neurotoxicity, and reduced treatment tolerance [18]. In contrast, localized drug delivery to the cervicovaginal region has the theoretical advantage of increasing drug exposure at the disease site while reducing systemic adverse effects; however, achieving prolonged retention, controlled release, mucosal compatibility, and patient-specific anatomical fitting remain technically challenging [19]. Finally, complex pelvic anatomy and multimodal treatment strategies are often difficult to communicate effectively using conventional two-dimensional imaging alone, creating unmet needs in surgical planning, radiotherapy training, patient education, and multidisciplinary decision-making [20]. These limitations collectively highlight the need for patient-centered, structurally controllable, and functionally adaptable platforms for cervical-cancer-related management.

Polymer-based additive manufacturing provides a promising strategy to address these unmet needs by integrating patient-specific design, digital fabrication, and material adaptability into a unified manufacturing workflow. As illustrated in Figure 1, this approach can convert clinical imaging or digital anatomical information into customized three-dimensional structures, allowing geometric adaptation to individual cervicovaginal and pelvic anatomy. Compared with conventional subtractive or mold-based fabrication, additive manufacturing enables layer-by-layer construction of complex architectures, including porous scaffolds, curved channels, anatomical replicas, and cell-laden hydrogel constructs [21,22]. More importantly, printable polymeric materials, including thermoplastic filaments [23], photocurable resins [24], biodegradable polymers [25], elastomers [26], and hydrogel bioinks [27], provide a broad material toolbox for balancing mechanical support, flexibility, biocompatibility, drug-loading capacity, and structural fidelity. These features make polymer-based additive manufacturing particularly suitable for cervical-cancer-related applications, including therapeutic scaffolds and local delivery systems, bioprinted research platforms, brachytherapy-assisting devices, and educational anatomical models. Therefore, rather than serving merely as a prototyping tool, polymer-based additive manufacturing may function as a patient-centered platform technology that connects clinical needs with structurally controllable and functionally adaptable biomedical devices.

Given these advantages, a dedicated review of polymer-based additive manufacturing in cervical-cancer-related management is timely and necessary. Existing reviews have primarily focused on specific clinical domains, especially three-dimensional printing-assisted gynecological or cervical cancer brachytherapy [16,28,29], in which patient-specific applicators, vaginal templates, needle-guidance devices, and anatomical models have been summarized. Other reviews have discussed individualized three-dimensional printing in gynecology or oncology more broadly [30,31], but these studies were not designed to analyze cervical-cancer-specific applications from a polymer materials and additive manufacturing perspective. In parallel, emerging studies on cervical implant scaffolds, hydrogel bioinks, and bioprinted cervical tumor models suggest that polymer-based three-dimensional printing is expanding beyond radiotherapy assistance toward local therapeutic delivery and preclinical research platforms [32]. However, these directions have not yet been integrated into a unified framework that links printable polymeric materials, manufacturing processes, patient-specific structures, and clinical or translational functions in cervical cancer.

Therefore, this review aims to provide a comprehensive overview of polymer-based additive manufacturing for cervical-cancer-related applications. Specifically, as shown in Figure 1, we summarize four major application areas: therapeutic scaffolds and local delivery systems, bioprinted research platforms for cervical cancer modeling, brachytherapy-assisting devices, and patient-specific anatomical models for education, planning, and communication. By organizing the literature from the perspective of material–process–structure–function relationships, this review seeks to clarify the current status, clinical relevance, technical limitations, and future opportunities of polymer-based additive manufacturing in cervical cancer management. Unlike previous reviews that mainly focus on brachytherapy or general gynecological applications, this review emphasizes polymer-based additive manufacturing as a material-enabled platform technology for cervical-cancer-related management.

## 2. Polymer-Based Additive Manufacturing in Cervical Cancer Management

### 2.1. 3D-Printed Polymeric Materials for Brachytherapy Assistance in Cervical Cancer

#### 2.1.1. Clinical Application Scenarios and Limitations of Cervical Cancer Brachytherapy

Radiotherapy is a central component of cervical cancer treatment, and brachytherapy is particularly indispensable for achieving adequate local dose escalation in locally advanced disease and selected recurrent settings [33,34]. Unlike external beam radiotherapy, brachytherapy places radioactive sources in close proximity to the cervical tumor or recurrent pelvic lesion, thereby enabling steep dose gradients and improved sparing of adjacent organs at risk [35]. However, the clinical implementation of cervical cancer brachytherapy is strongly constrained by patient-specific pelvic anatomy, tumor geometry, vaginal conditions, and the spatial relationship between the target volume and surrounding organs.

In locally advanced cervical cancer, intracavitary brachytherapy alone may be insufficient for bulky tumors, asymmetric residual disease, parametrial extension, or irregular target geometry [36]. Hybrid intracavitary/interstitial brachytherapy can improve target coverage by adding interstitial needles to conventional applicators, but accurate needle placement requires considerable procedural experience, image-guidance proficiency, and reliable control of needle angle, depth, and insertion point. Commercial applicators are usually available only in limited standard sizes and predefined channel configurations, which may restrict their applicability in patients with narrow vaginal anatomy, distorted pelvic anatomy, or tumors requiring individualized oblique or non-coplanar needle trajectories [37].

These limitations are even more pronounced in recurrent cervical cancer. Non-central recurrent lesions are frequently located deep in the pelvis and may be adjacent to major vessels, nerves, bowel, bladder, rectum, bone, or the pelvic sidewall. In such cases, conventional freehand interstitial implantation is technically demanding because the spatial distribution of needles, including distance, depth, and angle, is difficult to reproduce. Deviation between preoperative planning and actual needle placement may compromise target coverage or increase exposure to organs at risk. Moreover, repeated intraoperative imaging may be required to correct needle position, increasing procedural complexity, operation time, patient discomfort, and potential perioperative risk [38]. Therefore, cervical cancer brachytherapy represents a treatment field in which patient-specific geometry, reproducible needle guidance, and material-compatible device fabrication are clinically important.

#### 2.1.2. 3D-Printed Polymeric Devices as Patient-Specific Solutions

Polymer-based additive manufacturing provides a practical strategy to translate imaging-based treatment plans into physical guidance devices. In cervical cancer brachytherapy, 3D-printed polymeric templates and applicator accessories can transform virtual needle trajectories into patient-specific channels, thereby improving geometric adaptation, needle reproducibility, procedural efficiency, and target dose coverage [37]. These devices are typically fabricated from biocompatible, sterilizable, and imaging-compatible polymeric materials, including medical photopolymer resins and other printable polymer systems. Their relatively low cost, rapid production, and flexible design make them suitable for individualized treatment workflows. However, several limitations remain. Patient-specific fabrication depends on accurate image segmentation, device design, printing, and quality control, which may increase workflow complexity and require specialized technical expertise. In addition, material-specific properties, including sterilization tolerance, dimensional stability, imaging compatibility, and radiation-related safety, require careful validation before routine clinical use. Current clinical evidence is also derived largely from single-center studies or relatively small cohorts, and whether these devices improve long-term oncological outcomes remains uncertain.

**Figure 2:**
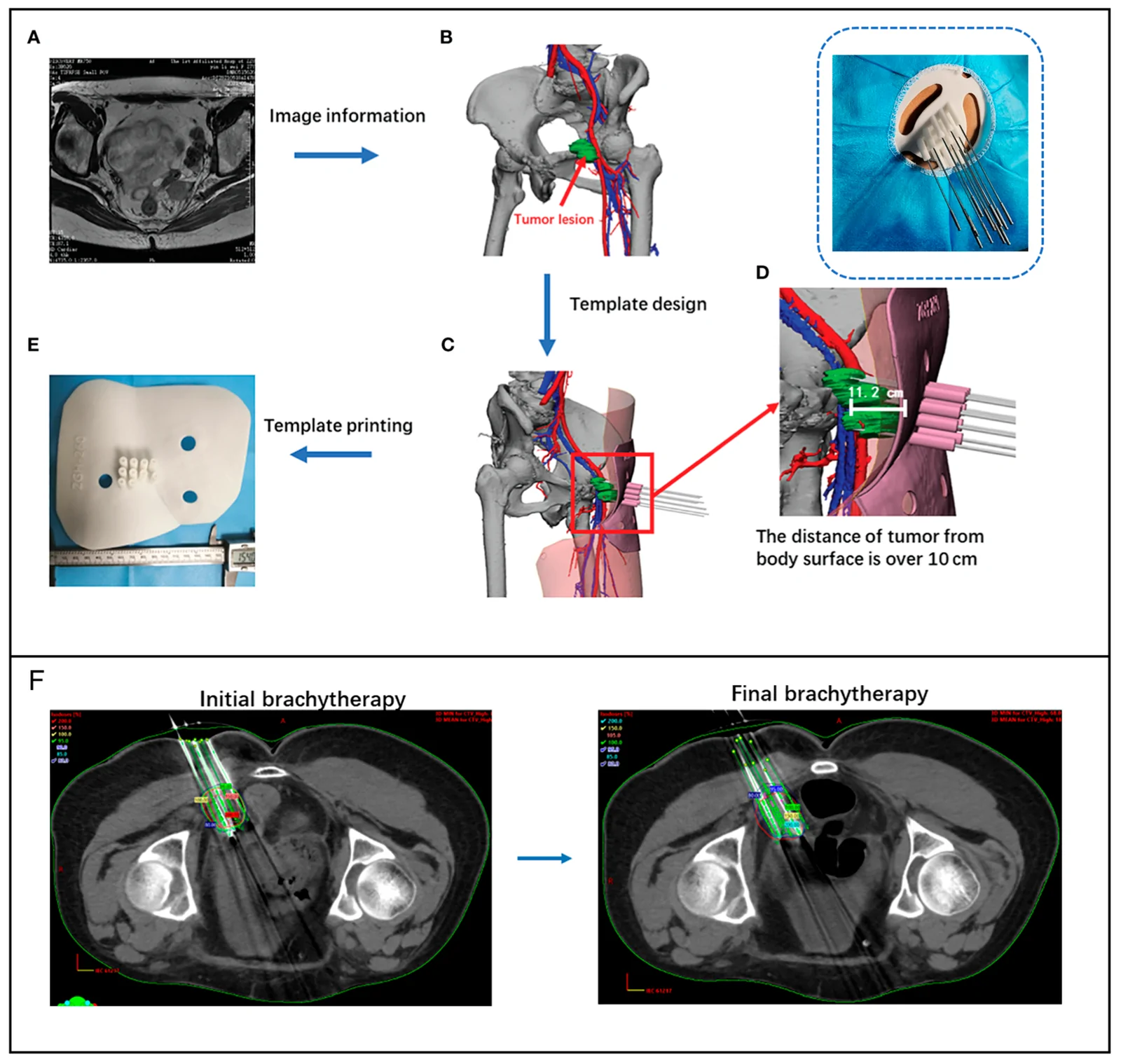
(**A**–**E**) Workflow of 3D-printed template-assisted interstitial brachytherapy for recurrent cervical cancer. Representative workflow of patient-specific 3D-printed non-coplanar template design and template-guided needle implantation, including preoperative imaging, target and organ-at-risk delineation, template design, physical template fabrication, and needle placement. (**F**) Comparison between preoperative and postoperative dosimetric plans, showing the role of 3D-printed templates in translating virtual treatment planning into reproducible clinical implantation [38].

A representative example is the application of a three-dimensional (3D)-printed non-coplanar template (3D-PNCT) for high-dose-rate (HDR) interstitial brachytherapy (ISBT) in non-centrally recurrent cervical cancer (NRCC). NRCC is particularly suitable for illustrating the clinical value of polymer-based template guidance because recurrent lesions are often located deep in the pelvis and adjacent to critical vessels, nerves, bones, bowel, bladder, or rectum [38]. In this situation, freehand implantation is limited by the difficulty of accurately controlling needle entry points, insertion depth, puncture angle, and spatial distribution. Figure 2A–E illustrates how imaging information was converted into a patient-specific 3D-printed guidance device. Figure 2A shows the preoperative magnetic resonance imaging (MRI) image used to identify the recurrent pelvic lesion. Based on the imaging data, Figure 2B reconstructs the pelvic anatomy and highlights the tumor lesion in relation to surrounding bones and major vessels, emphasizing the anatomical complexity of non-central pelvic recurrence. Figure 2C further translates this anatomical model into a template design, in which the planned non-coplanar needle trajectories are arranged to reach the target while avoiding adjacent critical structures. Figure 2D provides an enlarged view of the recurrent lesion and shows that the tumor was located more than 10 cm from the body surface, explaining why simple surface puncture or conventional transvaginal guidance may be insufficient. Finally, Figure 2E presents the physical 3D-PNCT, in which multiple guide channels and surface-fitting features convert the virtual preplan into a reproducible operative tool. The dosimetric relevance of this workflow is shown in Figure 2F. The comparison between the initial and final brachytherapy plans demonstrates that the template-guided implantation maintained a similar spatial relationship between the inserted needles, target volume, and isodose lines during treatment. In Wang et al.’s cohort of 36 NRCC patients, the postoperative dosimetric parameters were generally consistent with the preoperative plan, and no serious perioperative complications or grade 3–4 acute radiotherapy toxicities were observed [38]. Therefore, this study does more than demonstrate technical feasibility: it shows how a 3D-printed polymeric template can function as a physical interface between imaging-defined tumor geometry, preplanned needle trajectories, and clinically reproducible HDR-ISBT delivery.

For locally advanced cervical cancer, individualized vaginal templates represent another important application. Lindegaard et al. reported an individualized 3D-printed vaginal template for magnetic resonance imaging (MRI)-guided intracavitary/interstitial brachytherapy. This early technical work showed that customized vaginal templates could overcome limitations of standard applicator size and fixed needle paths, especially in anatomically constrained patients. Although the study was presented as a technical workflow rather than a large clinical series, it established an important concept: 3D printing can generate low-cost, patient-adapted applicator components for cervical cancer brachytherapy [37].

More recent studies have attempted to standardize and simplify template-assisted needle guidance. Marar et al. developed tandem-anchored radially guiding interstitial templates (TARGITs) for tandem-and-ovoid (T&O) HDR brachytherapy [39]. By attaching a 3D-printed, biocompatible and sterilizable template to standard tandem applicators, the TARGIT system guided interstitial needles toward planned dosimetric positions. Compared with non-template procedures, TARGIT improved high-risk clinical target volume (HR-CTV) coverage without increasing dose to nearby organs at risk (OARs) [39]. The same group later introduced TARGIT-Flexible-eXtended (TARGIT-FX), a next-generation design with extended access and greater flexibility for needle insertion. TARGIT-FX improved tumor dosimetry, shortened procedure time, and was considered easier to use by trainees, suggesting that 3D-printed devices may also lower the technical barrier of advanced intracavitary/interstitial procedures [40].

Clinical comparative evidence also supports the procedural value of 3D-printed brachytherapy. Lu et al. compared 3D-printed brachytherapy with freehand implantation in cervical cancer patients and reported improved dose distribution and reduced radiation exposure to critical organs, particularly in patients with large tumors or complex anatomical structures. Although survival benefits remain unproven, these findings suggest that 3D-printed guidance may improve treatment precision and safety rather than simply serving as an anatomical model [41].

Finally, 3D printing enables more complex needle geometries that are difficult to achieve using conventional applicators. Zhang et al. developed an individual curved-needle interstitial template (ICIT) for distal parauterine or vaginal cuff tumor recurrence [42]. This design addressed the limitation of straight or fixed needle channels when recurrent lesions are far from the vaginal wall. By designing curved needle paths according to computed tomography (CT), MRI, HR-CTV geometry, and vaginal filling models, the authors demonstrated the feasibility of curved-needle HDR brachytherapy with acceptable target and OAR dose parameters [42]. This work expands the role of 3D printing from simple template fabrication to individualized spatial route design.

Taken together, these studies suggest that 3D-printed polymeric brachytherapy devices can address several key limitations of conventional cervical cancer brachytherapy. First, they improve anatomical adaptation by matching applicator geometry to individual cervicovaginal or pelvic anatomy. Second, they convert planned needle trajectories into physical guide channels, improving reproducibility of needle angle, depth, and spatial distribution. Third, they may improve HR-CTV coverage and OAR sparing in bulky, asymmetric, parametrial, recurrent, or anatomically complex lesions. Fourth, their use of printable polymeric materials is clinically relevant because these materials can be lightweight, low-cost, sterilizable, biocompatible, and compatible with CT- or MRI-based workflows. Therefore, polymer-based 3D printing should be viewed not merely as a manufacturing method, but as a clinically meaningful interface between imaging-defined tumor geometry and individualized radiation delivery.

### 2.2. 3D-Printed Therapeutic Scaffolds for Cervical Cancer

#### 2.2.1. Clinical Application Scenarios and Limitations of Therapeutic Scaffolds for Cervical Cancer

Scaffold-based strategies have emerged as a potential adjunct to conventional cervical cancer treatment, particularly in scenarios where local therapy, tissue support, and sustained drug exposure are required [43]. In contrast to systemic chemotherapy or externally applied topical formulations, scaffolds can serve as localized therapeutic platforms that remain in contact with cervicovaginal tissue and provide spatially confined biological or mechanical functions [44]. Their applications in cervical-cancer-related treatment can be broadly divided into three categories: post-surgical tissue support, local drug delivery, and functional mucosal interface regulation.

Polymeric materials are especially suitable for these scaffold-based applications because their physicochemical properties can be adjusted over a broad range [45,46]. Biodegradable polyesters, elastomeric polyurethanes, polysaccharide derivatives, hydrogels, and bio-adhesive polymers can be engineered to provide different levels of flexibility, porosity, degradation behavior, swelling capacity, mucoadhesion, and drug-release kinetics [47,48]. These features are highly relevant to the cervicovaginal environment, where tissue softness, mucus turnover, fluid drainage, acidic pH, and anatomical variability can strongly influence device retention and therapeutic performance [49].

First, cervical scaffolds may provide structural support after local excisional procedures such as conization [43]. Removal of cervical tissue can create a local defect and may compromise cervical mechanical integrity, especially in patients with fertility-preservation needs. Patient-specific polymeric scaffolds therefore offer a possible route to restore local geometry, support the defect site, and maintain intimate contact with cervical tissue after surgery. Second, scaffolds can function as drug-eluting platforms for local delivery of antiviral, anticancer, immunomodulatory, or radio-sensitizing agents. This is particularly relevant for HPV associated cervical lesions, residual microscopic disease, and postoperative recurrence prevention, where prolonged local exposure may be more desirable than repeated systemic administration [19,50]. Third, scaffold-like polymeric systems, including porous implants, hydrogels, and bio-adhesive films, can improve residence time and tissue adherence in the cervicovaginal region, thereby compensating for the limited retention of conventional liquid or semisolid formulations [51].

#### 2.2.2. Research in 3D-Printed Polymeric Therapeutic Scaffolds

3D printing further expands the therapeutic potential of polymeric cervical scaffolds by allowing geometry, porosity, mechanical properties, and drug-loading patterns to be designed according to patient anatomy and treatment goals [52]. Instead of acting as passive fillers, 3D-printed polymeric scaffolds can integrate anatomical adaptation, mechanical compliance, mucosal compatibility, and controlled release within a single construct. However, this field remains at an early translational stage [53]. Current studies mainly support feasibility in personalized cervical implants, nanoparticle-releasing hydrogels, and printed bio-adhesive drug-delivery systems, while robust clinical evidence for long-term safety and therapeutic benefit is still lacking. Therefore, scaffold-based approaches should currently be viewed as experimental polymer-enabled local therapy platforms that may complement, rather than replace, established cervical cancer treatments.

The current research on 3D-printed polymeric therapeutic scaffolds for cervical-cancer-related applications remains limited, but several representative directions have already emerged. These studies can be broadly classified into patient-specific cervical implants for post-conization tissue support, drug-loaded scaffolds for prolonged antiviral delivery, nanoparticle-eluting hydrogels for local oncological therapy, and printed bio-adhesive films for mucosal drug retention.

A first research direction focuses on patient-specific cervical implants designed to restore local geometry and provide mechanical support after conization. Zhao et al. developed a personalized cervical tissue implant with protein-release function using polyurethane and low-temperature deposition manufacturing [43]. The implant was designed as a cone-shaped porous structure according to the depth and geometry of cervical conization. The use of polyurethane was important because this elastomeric polymer could provide flexibility and mechanical compliance closer to soft cervical tissue, while the porous architecture increased the available surface area for protein loading and release. In this system, anti-HPV protein was loaded into the implant after printing. The study demonstrated the feasibility of combining anatomical adaptation, soft-tissue support, and local antiviral protein delivery within a single printed construct. However, the protein was mainly loaded by post-printing adsorption, leading to rapid release within a short time window, which limited its ability to meet longer therapeutic requirements.

To address this limitation, Ji et al. further developed 3D-printed cervical implant scaffolds incorporated with drug-loaded carboxylated chitosan microspheres for long-term anti-HPV protein delivery [54]. In this design, polyurethane still served as the main structural scaffold, while carboxylated chitosan microspheres were introduced as functional drug reservoirs. Compared with directly mixing or adsorbing protein onto the scaffold, microsphere-based loading helped protect protein bioactivity and prolonged release. The authors also compared different incorporation strategies, including microsphere embedding and microsphere surface modification. This work is important because it shifts the scaffold from a simple porous carrier toward a composite polymeric delivery system, in which the structural polymer determines the macroscopic support and the polysaccharide microspheres regulate the protein-release behavior. From a materials perspective, this design illustrates how combining an elastomeric scaffold with bioactive polysaccharide-based microspheres can decouple mechanical support from drug-release control.

**Figure 3:**
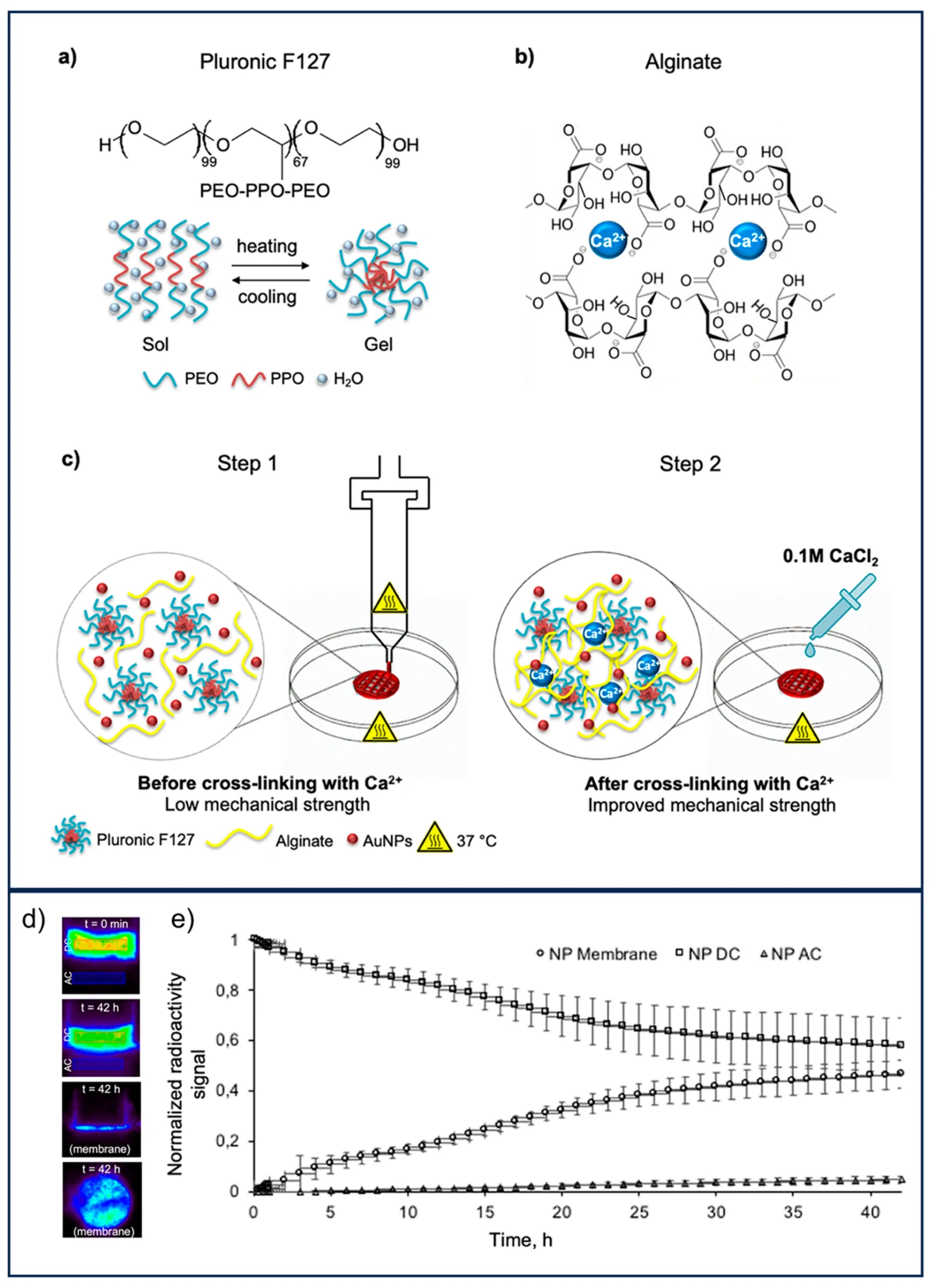
Design, fabrication, and local AuNP delivery of a 3D-printable Pluronic F127/alginate hydrogel, adapted from [55]. (**a**) Thermoresponsive sol–gel transition of Pluronic F127. (**b**) Ca^2+^-mediated alginate crosslinking. (**c**) Extrusion printing of the AuNP-loaded hydrogel followed by Ca^2+^-induced stabilization. (**d**) Representative PET images showing AuNP distribution and vaginal membrane accumulation over 42 h. (**e**) Time-dependent radioactivity signals in the donor compartment (DC), acceptor compartment (AC), and vaginal membrane, indicating AuNP release, mucosal retention, and limited transmembrane transport.

A second strategy involves 3D-printable hydrogels for localized nanoparticle delivery. Kiseleva et al. developed an AuNP-loaded hydrogel based on Pluronic F127 and alginate for cervical-cancer-related local therapy [55]. The two polymers provide complementary functions: Pluronic F127 enables thermoresponsive sol–gel transition near physiological temperature, whereas alginate allows Ca^2+^-mediated ionic crosslinking and improves structural stability. As illustrated in Figure 3a–c, the AuNP-containing formulation can be extrusion-printed into a defined geometry and subsequently stabilized by calcium-induced alginate crosslinking. This combination integrates printability, thermoresponsive gelation, structural fixation, and local nanoparticle delivery within a single polymeric system.

In addition to demonstrating printability, the study evaluated AuNP release and transport across porcine vaginal mucosa using PET imaging. The representative images and time-dependent radioactivity profiles in Figure 3d,e show progressive release from the hydrogel, pronounced accumulation within the vaginal membrane, and comparatively limited transport into the acceptor compartment. These findings suggest that the system favors local mucosal retention rather than extensive transmembrane diffusion, an advantageous feature for localized nanoparticle-based treatment. Nevertheless, the evidence remains primarily preclinical, and further studies are required to establish in vivo retention, safety, therapeutic efficacy, and the reproducibility of patient-specific printed constructs.

A third group of studies used printing technology to fabricate bio-adhesive drug-delivery films for cervical administration. Varan et al. first reported inkjet printing of antiviral polycaprolactone (PCL)-based nanoparticles and anticancer cyclodextrin inclusion complexes onto a bio-adhesive hydroxypropyl-cellulose film for cervical administration [56]. Paclitaxel was complexed with cyclodextrin to overcome poor aqueous solubility, while cidofovir was encapsulated in PCL or poly(ethylene glycol)-polycaprolactone nanoparticles to enable controlled antiviral release. Although this system is better described as a printed bioadhesive film rather than a true 3D scaffold, it is highly relevant to scaffold-like mucosal platforms because it demonstrates dose and geometry control on a polymeric substrate. In a follow-up study, the same group performed mechanical and ex vivo evaluation of these printed films [57]. Inkjet printing did not negatively affect film thickness and increased mechanical strength and surface free energy; the printed anticancer formulation was retained on sheep cervix tissue for more than 12 h. These findings support the broader concept that polymeric mucosal platforms can be engineered not only for drug loading, but also for adhesion, mechanical integrity, and local residence.

Taken together, these studies indicate that therapeutic scaffold-like systems for cervical cancer are moving in three complementary directions. Rigid or semi-flexible porous implants are being explored for post-conization tissue support and local anti-HPV delivery [43,54]. Composite polymeric scaffolds incorporating microspheres are being developed to prolong protein release while preserving bioactivity [54]. Printable hydrogels and bio-adhesive films are being used to improve mucosal contact, nanoparticle retention, and spatial control of drug administration [55,56,57]. Across these systems, the central advantage of polymers lies in their tunable structure–property relationships: elasticity can be matched to soft cervical tissue, porosity can regulate protein loading and cell contact, hydrophilicity and swelling can influence mucosal interaction, and degradation or diffusion properties can shape release kinetics. However, the field is still at an early proof-of-concept stage. Most studies remain in vitro or ex vivo, and there is still limited evidence regarding in vivo retention, long-term mucosal safety, reproductive outcomes, degradation products, sterilization stability, and clinical efficacy. Future work should therefore move from material feasibility toward clinically relevant validation models and standardized performance metrics.

### 2.3. Bioprinted Research Platforms for Cervical Cancer Modeling

#### 2.3.1. Research Application Scenarios and Limitations of Conventional Cervical Cancer Models

Beyond direct therapeutic devices, polymer-based additive manufacturing has also been used to construct in vitro research platforms for cervical cancer modeling. In this context, a research platform refers to an engineered experimental model designed to reproduce selected features of cervical tumor tissues under controllable laboratory conditions. These platforms are mainly used for studying tumor cell behavior, tumor–matrix interactions, drug response, epithelial–mesenchymal transition (EMT), hypoxia, molecular diffusion, and other microenvironment-dependent biological processes [58,59]. They are therefore distinct from implantable scaffolds or radiotherapy-assisting devices: their primary role is not clinical intervention, but disease modeling and preclinical evaluation.

The need for such platforms arises from the intrinsic limitations of conventional cervical cancer models. Two-dimensional cell culture remains useful for basic molecular assays, but it poorly represents the spatial architecture of tumor tissue. Cells grown as monolayers lack the three-dimensional organization, cell–matrix interactions, diffusion barriers, oxygen and nutrient gradients, and multicellular spheroid formation that shape tumor behavior in vivo [60,61]. Consequently, two-dimensional models may underestimate or distort key cancer phenotypes, including proliferation, invasion-related protein expression, EMT response, and chemoresistance. Animal models provide higher biological complexity, but they are limited by interspecies differences, ethical constraints, cost, low throughput, and reduced control over local matrix composition and tumor geometry [62].

Three-dimensional spheroids, hydrogel embedding, porous scaffolds, and microfluidic systems have partly addressed these limitations by introducing spatial structure and matrix-associated signaling [63]. However, these approaches often remain constrained by limited control over construct geometry, cell distribution, spheroid size, and reproducibility [64]. These parameters are not merely technical details, because tumor size and architecture directly influence oxygen availability, metabolic gradients, drug penetration, and treatment response. Therefore, cervical cancer modeling requires a platform that is both biologically more relevant than monolayer culture and more structurally controllable than conventional three-dimensional culture systems.

3D bioprinting provides such an intermediate strategy by enabling the deposition of cervical cancer cells and polymeric bioinks into predefined three-dimensional constructs. By combining living cells with hydrogel matrices such as alginate, gelatin, fibrinogen, Matrigel, hydroxyethylcellulose, and related polymeric systems, bioprinting allows cervical tumor-like models to be fabricated with controlled architecture and experimentally adjustable matrix properties [32,64,65,66,67]. This makes 3D-bioprinted cervical cancer models particularly suitable for investigating how tumor cells respond to a defined three-dimensional microenvironment, before moving toward more complex in vivo or patient-derived validation systems.

#### 2.3.2. Research Progress in 3D-Bioprinted Cervical Cancer Models

The first representative attempt to construct a 3D-bioprinted cervical tumor model was reported by Zhao et al., who printed HeLa cells within a gelatin/alginate/fibrinogen hydrogel system to generate an in vitro cervical tumor-like construct [32]. This study is important because it established the basic material–cell–printing logic for subsequent cervical cancer bioprinting work. Gelatin, alginate, and fibrinogen were selected as extracellular matrix-mimicking hydrogel components: gelatin contributed thermoresponsive processability and cell-interactive protein-derived cues, alginate provided ionic crosslinking and construct stabilization, and fibrinogen introduced a fibrin-like matrix component after thrombin-mediated crosslinking. The resulting bioink allowed HeLa cells to be directly incorporated into a printable hydrogel matrix and arranged into a designed three-dimensional grid structure.

**Figure 4:**
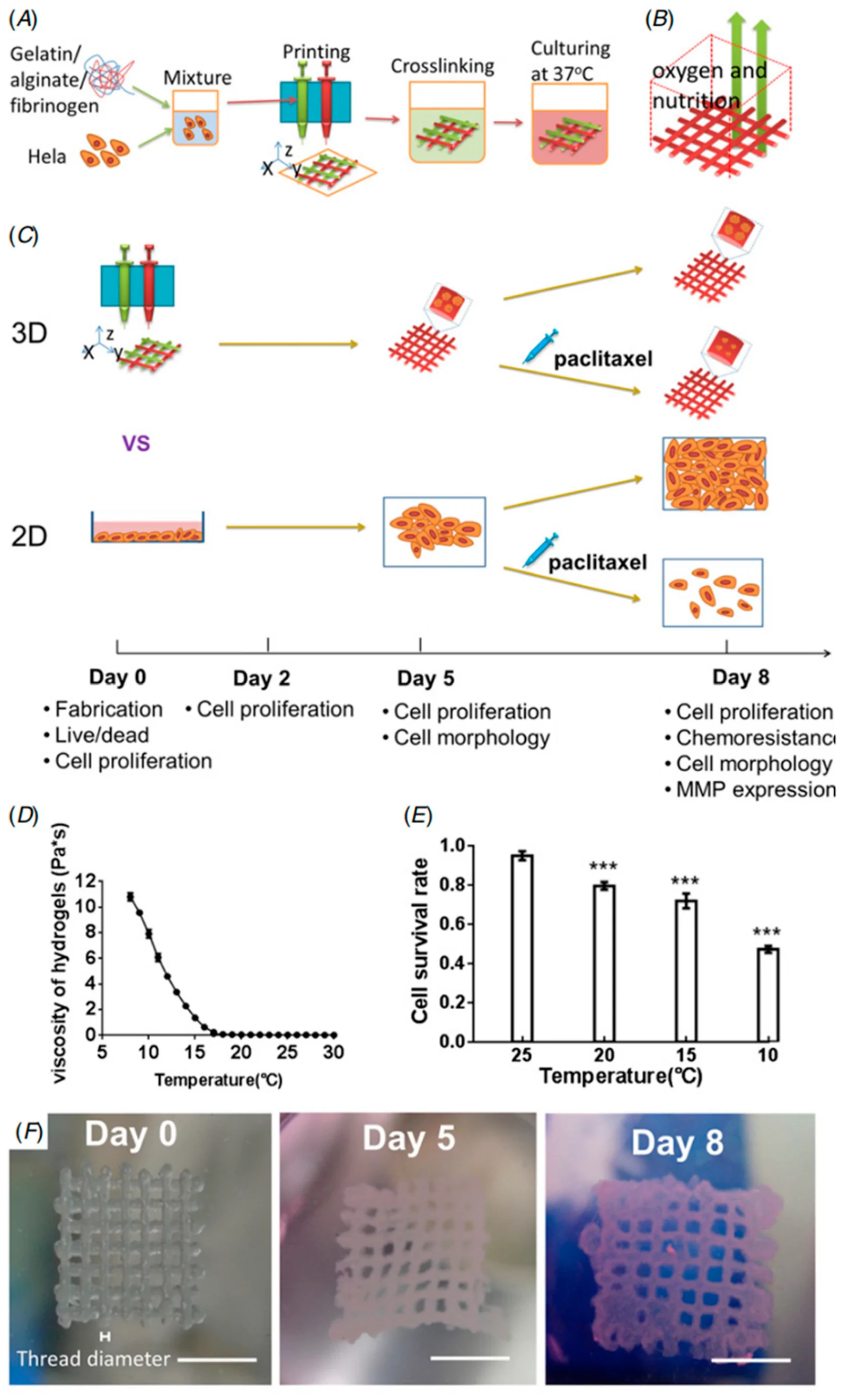
Fabrication and process optimization of a 3D-bioprinted HeLa cervical tumor model, adapted from [32]. (**A**) Extrusion-based bioprinting workflow in which HeLa cells were mixed with gelatin/alginate/fibrinogen hydrogel, deposited into a predefined grid-like construct, crosslinked, and cultured at 37 °C. (**B**) Design of the interconnected grid architecture for supporting oxygen and nutrient transport within the printed construct. (**C**) Experimental timeline comparing 3D-bioprinted HeLa/hydrogel constructs with conventional two-dimensional culture, including assessment of cell proliferation, morphology, chemoresistance, and matrix metalloproteinase expression, with paclitaxel treatment introduced during the later culture period. (**D**) Temperature-dependent viscosity of the HeLa/hydrogel bioink. (**E**) Effect of printing temperature on post-printing cell survival. (**F**) Representative macroscopic images of the printed constructs on days 0, 5, and 8, showing maintenance of the grid-like architecture during culture. Scale bars: 5 mm.

This study systematically connected material preparation, printing optimization, and biological validation. Figure 4A illustrates the overall fabrication process, in which HeLa cells were mixed with gelatin, alginate, and fibrinogen; extruded into a predefined construct; and subsequently stabilized through sequential crosslinking. Figure 4B shows the designed architecture of the printed cervical tumor model, emphasizing a grid-like geometry rather than a bulk hydrogel block. This design was functionally relevant because the interconnected channels could facilitate nutrient transport, oxygen exchange, and metabolic waste removal during culture. Figure 4C presents the experimental timeline, in which the printed 3D constructs and two-dimensional controls were cultured in parallel and then evaluated with or without paclitaxel exposure. This layout enabled direct comparison between conventional monolayer culture and the printed 3D tumor model. Figure 4D shows the temperature-dependent viscosity of the hydrogel formulation. Because gelatin-containing hydrogels become more viscous at lower temperature, printing temperature directly affected extrusion behavior and structural fidelity. Figure 4E further links this processing variable to cell survival, showing that lower nozzle temperature reduced post-printing viability, probably due to increased viscosity and shear stress during extrusion. Therefore, Figure 4D,E together demonstrate that bioink rheology and printing parameters must be balanced rather than optimized independently. Finally, Figure 4F shows the macroscopic appearance of the printed constructs during culture. The constructs maintained a recognizable grid structure from the initial stage to later culture time points, supporting the feasibility of using this hydrogel-based printed system as a stable cervical tumor model [32].

Biologically, the printed 3D model produced behaviors that were not reproduced by standard two-dimensional culture. HeLa cells maintained high post-printing viability, proliferated within the hydrogel matrix, and gradually formed multicellular spheroids. Compared with monolayer culture, cells in the printed 3D constructs showed stronger proliferative activity, increased matrix metalloproteinase expression, and higher resistance to paclitaxel treatment [32]. These findings indicate that 3D bioprinting did not merely change the physical format of culture, but altered tumor-relevant phenotypes by providing a spatially confined hydrogel microenvironment. From a cervical cancer modeling perspective, this study provided the first proof-of-concept that polymeric bioinks could be used to fabricate reproducible HeLa-based tumor constructs with spheroid formation, invasion-associated protein expression, and drug-response differences.

Building on this foundation, Pang et al. further used a 3D-printed HeLa/hydrogel model to investigate TGF-β-induced epithelial–mesenchymal transition [64]. In this study, HeLa cells were printed in a composite hydrogel containing gelatin, alginate, and Matrigel. The printed grid construct supported rapid cell proliferation and spheroid formation. After TGF-β stimulation, the spheroids began to lose their compact epithelial-like organization, and some cells acquired a spindle-like morphology. Molecular analyses confirmed downregulation of the epithelial marker E-cadherin and upregulation of mesenchymal markers including Snail, vimentin, and N-cadherin. Importantly, the EMT response differed between 3D and 2D systems, suggesting that matrix embedding, spheroid organization, and diffusion-limited exposure to soluble factors can modulate EMT signaling. The authors also showed that disulfiram and the EMT pathway inhibitor C19 attenuated TGF-β-induced EMT in this model [64]. This work expanded the function of 3D-bioprinted cervical cancer models from phenotypic comparison to mechanistic interrogation of tumor invasion and metastasis-related programs.

Subsequent studies shifted greater attention toward bioink formulation and construct design. Gospodinova et al. developed hydroxyethylcellulose/sodium alginate bioinks for extrusion bioprinting of HeLa-based cervical tumor constructs [65]. This work was less focused on a specific oncogenic pathway and more focused on the material requirements for reliable bioprinting. By varying the relative alginate content, the authors correlated rheological behavior, printability, cell viability, residence time in the bioink, and post-printing proliferation. The study showed that HeLa cell viability was strongly affected by extrusion-related stress and by the viscosity of the bioink under printing-relevant shear conditions. A formulation containing hydroxyethylcellulose with lower alginate content supported better post-printing viability and subsequent proliferation, whereas excessive alginate compromised cytocompatibility [65]. This study is valuable because it clarified a central design rule for cervical tumor bioprinting: a bioink must provide sufficient viscosity and stability for printing, but excessive resistance to extrusion can damage cells and reduce model quality.

Othman et al. introduced another strategy by printing preformed HeLa spheroids rather than only dispersed single cells [66]. The authors prepared alginate–gelatin bioinks and optimized a multilayer hexagonal scaffold loaded with HeLa microtissues. The use of gelatin was intended to provide protein-derived cell-supportive cues, whereas alginate contributed printability and calcium-mediated stabilization. Compared with single-cell encapsulation, spheroid loading can preserve stronger cell–cell interactions and reduce direct exposure of isolated cells to shear stress during printing. The optimized alginate–gelatin formulation supported high-fidelity printing of multilayer constructs, and the embedded HeLa microtissues maintained high viability after culture. This work is conceptually important because it moved cervical cancer bioprinting from “printing cells that later form spheroids” toward “printing pre-assembled tumor microtissues into a designed scaffold,” which may better preserve multicellular architecture at the beginning of the experiment.

Most recently, Becconi et al. advanced 3D-bioprinted cervical tumor modeling by combining alginate-based bioprinting with nano-electrochemical characterization [67]. In this study, HeLa cells were bioprinted in an alginate-based matrix supplemented with mannitol, and the formulation was optimized to improve filament uniformity and post-crosslinking shape stability. The printed system generated spheroids over time and allowed evaluation of spheroid size, cellular organization, E-cadherin expression, oxygen concentration, and molecular diffusion. A particularly important aspect of this work was the use of scanning electrochemical microscopy with platinum nanoelectrodes to measure oxygen gradients inside HeLa spheroids and quantify the diffusion of a model molecule through the three-dimensional matrix [67]. This moved the field beyond conventional assessments of morphology and cell viability toward quantitative mapping of microenvironmental parameters [67]. For cervical cancer drug-screening platforms, such information is crucial because oxygen availability and molecular diffusion directly influence hypoxia-associated biology, therapeutic penetration, and apparent drug sensitivity.

Taken together, these studies demonstrate a clear progression in 3D-bioprinted cervical cancer modeling. The field began with proof-of-concept fabrication of HeLa/hydrogel constructs and comparison with two-dimensional culture [32], and then expanded toward EMT modeling and pathway inhibition [64], bioink rheology and cytocompatibility optimization [65], spheroid-laden scaffold construction [66], and finally spatially resolved analysis of oxygen and diffusion within the printed tumor microenvironment [67]. Across these studies, polymeric materials were not passive carriers. Their rheology determined printability and shear exposure; their crosslinking mechanisms stabilized the printed architecture; their stiffness and biochemical composition influenced cell survival, spheroid formation, and cell–matrix interaction; and their permeability regulated the transport of nutrients, oxygen, metabolites, and drug molecules. Therefore, the main advantage of polymers in 3D-bioprinted cervical cancer models lies in their tunable balance between processability and biological relevance. By adjusting polymer type, concentration, blending ratio, crosslinking strategy, viscosity, degradation behavior, and bioactive components, polymeric bioinks provide a controllable matrix framework for constructing reproducible cervical tumor models that are more physiologically informative than two-dimensional culture while remaining more experimentally tractable than animal models.

### 2.4. Patient-Specific Anatomical Models and Educational Guidance Tools

In addition to therapeutic devices and in vitro tumor models, polymer-based additive manufacturing has also been applied to patient-specific anatomical visualization and educational guidance in cervical cancer. This application is clinically relevant because cervical cancer management requires accurate understanding of the spatial relationship among the cervix, uterus, tumor mass, vaginal fornix, parametrial tissues, bladder, rectum, and pelvic sidewall. Although CT and MRI provide essential diagnostic information, their two-dimensional display may be difficult for patients, trainees, and even multidisciplinary teams to interpret intuitively. Physical 3D-printed models can therefore serve as tangible tools for surgical planning, procedural simulation, patient education, and interprofessional communication.

Baek et al. reported one of the earliest applications of patient-specific 3D printing in early-stage cervical cancer management [68]. In their case report, morphometric data were extracted from CT images to fabricate a physical model of the uterus, cervix, and tumor. The model was used during consultation with a young patient who initially wished to preserve fertility, and it helped her understand the tumor extent and treatment options before choosing radical hysterectomy. The model also supported surgical planning by helping the team evaluate tumor shape, location, and resection margins. This study showed that 3D-printed anatomical models may contribute not only to physician-side planning, but also to patient-centered decision-making.

The educational value of 3D printing was further illustrated by Bartellas et al., who developed a hemorrhagic cervical cancer model for postgraduate gynecological simulation training [69]. Unlike patient-specific surgical models, this model was designed as a reusable teaching tool for practicing clinical recognition and management of cervical tumor bleeding. Campelo et al. extended this concept to radiation oncology by developing a multi-material modular pelvic phantom for intracavitary and interstitial high-dose-rate brachytherapy training [70]. By simulating pelvic organs and cervical tumor conditions with different printed materials, the phantom provided a structured environment for teaching applicator placement and interstitial needle procedures without exposing patients to trainee-related procedural risk. More recently, Scherer-Quenzer et al. evaluated individualized 3D-printed cervical cancer models across FIGO stages I–III and reported their perceived value for examination, diagnosis, treatment planning, and multidisciplinary communication [71]. This study is important because it moved beyond single-case demonstration and assessed how different medical disciplines may use patient-specific models in gynecologic oncology.

Overall, 3D-printed anatomical and educational tools occupy a complementary position in cervical cancer management. They do not directly treat tumors and should not be overinterpreted as evidence-based therapeutic interventions. Their value lies in improving spatial comprehension, procedural training, treatment discussion, and communication among clinicians and patients. From a materials perspective, printable polymers and resins are particularly suitable for this application because they enable low-cost fabrication, rapid model production, color-coded anatomy, variable stiffness, and repeatable training models. Future studies should focus less on demonstrating visual feasibility and more on measurable outcomes, such as training performance, decision quality, patient understanding, procedural accuracy, workflow efficiency, and cost-effectiveness.

## 3. Conclusions and Perspectives

### 3.1. Conclusions

Polymer-based additive manufacturing is emerging as a versatile enabling technology for cervical-cancer-related management. Current evidence indicates that its value is not limited to the fabrication of isolated devices, but extends across several interconnected application domains, including brachytherapy assistance, therapeutic scaffolds and local delivery systems, bioprinted cervical cancer research platforms, and patient-specific anatomical models for education, planning, and communication. Across these scenarios, the central contribution of additive manufacturing lies in its ability to translate imaging data, anatomical requirements, and material design into customized three-dimensional structures. This is particularly relevant to cervical cancer, where disease management is strongly affected by complex pelvic anatomy, heterogeneous tumor geometry, cervicovaginal tissue characteristics, and the need for precise local intervention.

In brachytherapy, 3D-printed polymeric devices have shown clear procedural relevance. Patient-specific templates, applicator accessories, and needle-guidance systems can convert preoperative imaging and treatment plans into physical tools that improve anatomical adaptation and needle-placement reproducibility. Printable polymers are well suited to this role because they can be lightweight, low-cost, structurally precise, sterilizable, and compatible with CT- or MRI-based workflows. Therefore, in this field, polymers mainly contribute through geometric fidelity, workflow adaptability, and procedural safety rather than through direct biological activity.

In therapeutic scaffold and local delivery systems, the function of polymers is more biologically active. Polyurethane implants, chitosan-based microsphere composites, Pluronic F127/alginate hydrogels, polycaprolactone-based nanoparticles, and bioadhesive cellulose-derived films illustrate how polymeric systems can be engineered to provide mechanical support, local retention, controlled release, mucoadhesion, swelling, degradation, and tissue-interface regulation. These properties are highly relevant to the cervicovaginal environment, where fluid drainage, mucus turnover, tissue softness, acidic pH, and anatomical variability can compromise conventional formulations. In this domain, polymer-based additive manufacturing provides a route to integrate anatomical conformity with localized therapeutic function.

In bioprinted cervical cancer models, polymeric bioinks serve as artificial extracellular matrix platforms. Alginate, gelatin, fibrinogen, Matrigel-containing hydrogels, hydroxyethylcellulose, and alginate–gelatin systems have been used to support HeLa cell printing, spheroid formation, EMT modeling, drug-response testing, and quantitative analysis of oxygen and molecular diffusion. Here, the polymer matrix is not merely a structural support; it defines printability, shear exposure, cell viability, stiffness, permeability, matrix interaction, and long-term culture behavior. These models remain simplified compared with patient tumors, but they offer controllable platforms for studying cervical cancer biology under defined three-dimensional conditions.

In anatomical models and educational guidance tools, polymer-based printing provides a low-risk and practical approach for improving visualization, training, and communication. Rigid and flexible resins, multi-material phantoms, and color-coded anatomical models can represent patient-specific tumor anatomy, pelvic organs, procedural pathways, and training scenarios. Their main value lies in spatial comprehension, patient-centered explanation, procedural rehearsal, and trainee education. Although these tools do not directly improve tumor control, they may support better decision-making and safer clinical workflows.

To facilitate cross-study comparison, the representative literature is systematically summarized in Table 1 according to application category, polymer composition, key contribution, major limitations, and translational stage. Collectively, these studies encompass four major cervical-cancer-related applications of polymer-based additive manufacturing: brachytherapy guidance, therapeutic scaffolds and local delivery, bioprinted tumor models, and anatomical or educational models. The available evidence reveals a clear difference in translational maturity among these applications. Brachytherapy-guidance devices have achieved the greatest clinical proximity, with several studies demonstrating feasibility, procedural reproducibility, and potential dosimetric benefits. In contrast, therapeutic scaffolds remain predominantly preclinical, while bioprinted cervical tumor models are still largely restricted to in vitro research. Anatomical and educational models have shown practical value for visualization, training, and clinical communication, but their impact on objective clinical outcomes remains insufficiently established. Collectively, these findings indicate that the major strength of polymer-based additive manufacturing lies in combining material tunability with patient-specific structural design, although further standardized validation is required before broader clinical translation.

### 3.2. Perspectives

Future development should move from feasibility demonstration toward application-specific validation:

For brachytherapy, the next priority is not simply printing more individualized templates, but establishing standardized image-to-print workflows, quality-control criteria, sterilization protocols, mechanical accuracy testing, and prospective clinical evaluation. Polymeric materials used for this purpose should be assessed not only for print fidelity, but also for biocompatibility, sterilization tolerance, dimensional stability, imaging compatibility, and radiation-related safety. Further integration with MRI-guided adaptive brachytherapy, automated treatment planning, and artificial-intelligence-assisted trajectory design may expand the role of 3D-printed polymeric devices in complex primary and recurrent cervical cancer.

For therapeutic scaffolds and local delivery systems, the key challenge is biological translation. Future polymeric scaffolds should be designed according to the specific clinical scenario rather than as generic drug carriers. Post-conization implants, cervicovaginal mucosal delivery systems, postoperative recurrence-prevention platforms, radiosensitizer depots, and immunomodulatory local delivery systems may require very different material properties. Important design parameters include elasticity, degradation rate, drug-loading method, release kinetics, mucoadhesion, swelling behavior, local immune compatibility, and reproductive safety. In this context, biodegradable and bioadhesive polymers, stimuli-responsive hydrogels, microsphere-loaded composites, and multi-layered scaffolds may provide more clinically relevant platforms than single-function materials.

For bioprinted research platforms, future progress should focus on improving biological representativeness. Current cervical cancer models are mostly based on HeLa cells and relatively simple hydrogel matrices. More advanced systems should incorporate patient-derived cervical cancer cells, organoid-like epithelial structures, cancer-associated fibroblasts, endothelial cells, immune components, extracellular matrix gradients, and controllable oxygen or drug-diffusion profiles. Polymer design will be central to this process because bioink stiffness, degradability, ligand presentation, porosity, and permeability directly affect tumor cell behavior. A major future direction is the construction of standardized yet customizable polymeric bioinks that can support disease modeling, drug screening, radiotherapy-response testing, and personalized treatment evaluation.

For anatomical and educational tools, broader implementation will depend on whether 3D-printed models can demonstrate measurable benefits. Future studies should assess objective outcomes such as trainee performance, procedural accuracy, planning time, patient understanding, informed consent quality, multidisciplinary communication, and cost-effectiveness. Multi-material polymer printing may allow models with more realistic tactile properties, flexible cervicovaginal anatomy, puncture-compatible tissues, bleeding simulation, and replaceable tumor modules. These models may be particularly useful in gynecologic oncology training, brachytherapy education, rare anatomy simulation, and patient-specific preoperative planning.

From a broader materials perspective, the future of polymer-based additive manufacturing in cervical cancer will likely depend on four directions. First, printable polymers must become more clinically standardized, with clearer information on sterilization, mechanical performance, degradation products, and regulatory classification. Second, multi-material and multi-functional printing should be used to integrate mechanical support, drug release, imaging visibility, and biological interaction within the same construct. Third, digital workflows should be linked more closely with clinical imaging, treatment planning systems, and patient-specific anatomical data. Fourth, translational studies should include clinically meaningful endpoints rather than only technical feasibility or morphological similarity.

In the long term, polymer-based additive manufacturing may support a more personalized model of cervical cancer management. A single patient-specific workflow could theoretically connect MRI- or CT-derived anatomy, individualized brachytherapy guidance, customized local drug delivery, ex vivo bioprinted tumor testing, and patient-specific educational models. This vision remains distant, but it illustrates the broader potential of polymers as adaptable, printable, and functionally tunable materials. The most realistic path forward is not to present 3D printing as a replacement for established cervical cancer treatment, but to develop polymer-based additive manufacturing as a complementary technology that improves precision, local functionality, experimental modeling, and clinical communication.

## Figures and Tables

**Table 1:** Comparison of representative polymer-based additive manufacturing studies for cervical-cancer-related applications.

Application	Polymer	Key Contribution	Limitations	Translational Stage	Refs.
**Brachytherapy guidance**	Not mentioned	Patient-specific vaginal template for MRI-guided IC/IS brachytherapy	Single-case technical evidence	Clinical feasibility	[37]
**-**	PA12 nylon	3D-PNCT reproduced planned needle trajectories in recurrent cervical cancer	No established survival benefit	Clinical cohort	[38]
**-**	Biocompatible sterilizable polymer	TARGIT/TARGIT-FX improved guided interstitial needle placement and procedural efficiency	Limited single-center validation	Clinical translational study	[39,40]
**-**	PLA	Patient-specific curved-needle template for difficult pelvic recurrence	Small clinical series	Clinical feasibility	[42]
**Therapeutic scaffold/local delivery**	Polyurethane	Personalized porous cervical implant for mechanical support and anti-HPV protein delivery	Relatively rapid protein release after adsorption	Preclinical	[43]
**-**	Polyurethane, chitosan	Microspheres prolonged anti-HPV protein release	Long-term in vivo validation lacking	Preclinical	[54]
**-**	Pluronic F127, alginate, AuNPs	Printable hydrogel for localized AuNP delivery and mucosal retention	Mainly in vitro/ex vivo evidence	Preclinical	[55]
**-**	Hydroxypropyl cellulose + PCL/PEG-PCL nanoparticles	Printed antiviral/anticancer formulations onto a bio-adhesive cervical film	2D-structure	Preclinical	[56,57]
**Bioprinted tumor model**	Gelatin + alginate + fibrinogen	Established an early HeLa-based 3D cervical tumor model	Simplified HeLa-only system	In vitro	[32]
**-**	Gelatin + alginate + Matrigel	Modeled TGF-β-induced EMT and its inhibition	Limited tumor-microenvironment complexity	In vitro	[64]
**-**	Hydroxyethylcellulose + sodium alginate	Linked bioink rheology with printability and cell viability	Viscosity increases cell injury	In vitro	[65]
**-**	Alginate + gelatin	Printed preformed HeLa spheroids in multilayer scaffolds	Simplified cell-line model	In vitro	[66]
**-**	Alginate + mannitol	Quantified oxygen gradients and molecular diffusion in printed tumors	Limited biological complexity	In vitro	[67]
**Anatomical/educational models**	Not mentioned	Patient-specific cervix–tumor model for planning and patient communication	Single-case evidence	Clinical case report	[68]
**-**	PLA	Modular pelvic phantom for brachytherapy training	Tunable stiffness and reusable simulation; educational rather than therapeutic validation	Simulation/educational	[70]
**-**	PLA + magnetic-iron composite	MRI-derived individualized models for multidisciplinary assessment	Low-cost and visually intuitive; small sample and mainly subjective evaluation	Clinical/educational evaluation	[71]

## Data Availability

The original contributions presented in the study are included in the article; further inquiries can be directed to the corresponding author.
